# Juvenile Parkinson Disease

**DOI:** 10.7759/cureus.5409

**Published:** 2019-08-17

**Authors:** Arsalan Anwar, Sidra Saleem, Aisha Akhtar, Sara Ashraf, Mirza Fawad Ahmed

**Affiliations:** 1 Neurology, University Hospitals Cleveland Medical Center, Cleveland, USA; 2 Neurology, University of Toledo, Toledo, USA; 3 Medicine, Sharif Medical and Dental College, Lahore, PAK; 4 Internal Medicine, Sharif Medical and Dental College, Lahore, PAK; 5 Internal Medicine, Lahore Medical and Dental College, Lahore, PAK

**Keywords:** juvenile parkinson disease, tremors, atp13a2 mutation, pyramidal symptoms, extrapyramidal symptoms

## Abstract

Juvenile Parkinson’s disease (JPD) is a rare movement disorder that presents before the age of 21 years. Kufor-Rekab syndrome (KRS) is one of the distinct types of JPD caused by the ATP13A2 mutation and inherited as an autosomal recessive. The pathogenesis of KRS is related to an interrelated metabolism of ATP13A2 with Mn+2 and Zn+2, bioenergetics of mitochondria, autophagy lysosomal dysfunction, and synuclein metabolism. Clinically, KRS has a variable phenotype and may present with pyramidal or extrapyramidal symptoms and cognitive impairment. Early diagnosis of KRS is important as most of these patients are levodopa-responsive and genetic counseling and screening is important for the whole family. We present a case of a 16-year-old boy who presented with tremors and walking difficulty. His physical examination showed an expressionless face, decrease in eye blink frequency, and slow vertical saccadic eye movements. His movements were slow. All laboratory investigations were normal, except the genetic study, which led to the diagnosis of KRS.

## Introduction

Parkinson's disease (PD) is a movement disorder caused by a degeneration in the basal ganglia. It has four cardinal symptoms consisting of tremor, rigidity, bradykinesia, and postural instability. The incidence of PD increases with age, with an incidence of 0.5 per 100,000 in the < 40-year age group compared to 13.4 per 100,000 in the overall age group [1]. Early-onset Parkinson’s disease (EOPD) is defined as the onset of PD symptoms before the age of 60 years. EOPD is further divided into juvenile Parkinson’s disease (JPD) if it the patient presents before the age of 21 and young-onset Parkinson disease (YOPD) if the onset is between 21 and 40 years of age [2]. 

Several genetic mutations have been identified as a cause of JPD. Kufor-Rekab syndrome (KRS) is one of the distinct types of JPD that is caused by the ATP13A2 mutation with an autosomal recessive pattern of inheritance. It is one of the rare diseases with less than 50 cases reported so far [3]. We attempt to report a case of KRS and highlight the importance of rare movement disorders, thereby justifying the need for high suspicion in the management of such patients.

## Case presentation

A 16-year-old adolescent boy presented in our neurology clinic with a history of tremors in the left hand for eight months, followed by difficulty in walking for three months. The tremors were only present during rest without any change in frequency. Similarly, no exaggerating or initiating factors were identified. For the last two years, the patient has experienced increased restlessness, sweating, and nervousness, especially during social gatherings. The patient was born to a consanguineous marriage and has no significant past medical or family history. The patient was prescribed propranolol but no improvement was seen. The patient had also gone under cognitive-behavioral therapy without any beneficial outcome.

On clinical examination, the Glasgow Coma Scale (GCS) was 15/15 (E4, V5, M5). His vitals were a blood pressure of 120/70, heart rate of 80 beats per minute, respiratory rate at 18 breaths per minute, and afebrile. He had an expressionless face with a decrease in eye blink frequency. Tremors were noticeable in his left hand while resting on the table. Cranial nerve examination showed slow vertical saccadic eye movements. His vision and funduscopic examination were normal. Slit-lamp examination showed no Kayser‐Fleischer ring. Limb examination showed a Grade 2 rigidity (Parkinson’s Disease Rating Scale, Part III (UPDRS‐III)) in the left upper and lower limbs with 5/5 power and +2 deep tendon reflexes (DTR). Slowness was observed during hand movement, finger tapping, and toe-tapping. During walking, the patient had difficulty maintaining his balance with swaying on the left side. The patient also had difficulty in writing and letters became progressively smaller. 

Multiple investigations were carried out that involved a complete blood profile, comprehensive metabolic panel, serum iron, copper, ceruloplasmin, and 24-hour urine copper levels, which appeared to be normal. Magnetic resonance imaging (MRI) of the brain and spinal cord, along with visual evoked potentials, were also normal. On the basis of his history, physical examination (tremor, rigidity, bradykinesia, mask-like face, micrographia, and upgaze palsy), and age of onset of symptoms, a clinical diagnosis of juvenile parkinsonism (JPD) was considered. Molecular screening of the ATP13A2 gene on the deoxyribonucleic acid (DNA) extracted was performed. Sequence analysis revealed a homozygous deletion on exon 22 that resulted in the replacement of serine with threonine, followed by a stop codon. This confirmed the diagnosis of autosomal recessive juvenile parkinsonism. The patient's parents consented for the testing of a gene mutation which showed a heterozygous mutation of ATP13A2 gene, confirming an autosomal recessive pattern of disease.

The patient was started on a low dose of carbidopa/levodopa, resulting in a gradual improvement in tremor and walking. After four months of follow-up, the patient experienced dyskinetic movements of tongue and foot due to a peak concentration of dose. We lowered the dose of levodopa and started the patient on pramipexole (the dopamine agonist). He was followed for a year after that and his symptoms were stable but a little difficulty in writing still persisted.

## Discussion

KRS is an autosomal recessive, juvenile-onset, levodopa-responsive parkinsonism that is characterized by the onset of extrapyramidal, pyramidal, and cognitive dysfunction. It was first identified in 1994 when five cases of a Jordanian family were reported living in Kufr Rakeb, hence gaining the name of the disease [4]. There were multiple genes identified in 2006 as a causative factor behind the KRS disorder. These gene defects could be autosomal dominant (VPS35, LRRK2, SNCA), autosomal recessive (PINK1, PRKN, PARK7, ATP13A2, SLC6A3, and FBX07), or X-linked (TAF1). The gene that has been identified in KRS is Parkin-9 or ATP13A2. ATP13A2 is involved in Mn2+ and Zn2+ metabolism, mitochondrial bioenergetics, and autophagy lysosomal dysfunction, resulting in a decrease in dopaminergic neurons. Also, ATP13A2 is related to synuclein metabolism, which is the major component in the pathogenesis of Lewy body dementia [5]. 

Clinically, KRS manifests as parkinsonian signs of tremor, bradykinesia, rigidity, and postural instability. In addition, slow saccadic eye movement, supranuclear gaze palsy, ataxia, partial or total paralysis of the legs, spasticity, dystonia, dyskinesia, and hyperreflexia. Neurodegeneration involving multiple organs is evidenced by the presence of pyramidal signs and cognitive impairment. Many patients first present with learning difficulties and intellectual disability, and it is the most common non-motor symptom. Other non-motor symptoms include anxiety, hyposmia or anosmia, panic attacks, and auditory and visual hallucinations [6]. 

KRS can be confused with many diseases, such as dopa-responsive dystonia (DRD), Wilson’s disease, a juvenile form of Huntington’s disease, Parkinson-plus syndromes, neuroacanthocytosis, and several toxic and metabolic causes of PD. The few cases that have been reported in the literature have been delayed in diagnosis due to the variability of presentation [7]. Jha et al. reported a case of a 12-year-old male who presented with jerky movements of the left leg that progressed to generalized weakness and made the patient bedridden [8]. After five years, he was diagnosed with juvenile PD and responded to levodopa. Prashanth et al. reported a case of an 18-year-old boy who presented with hand tremors and difficulty in balance and responded to levodopa [9]. Suleiman et al. reported a case of compound heterozygosity of the novel mutation in ATP13A2 [10]. He presented with gait disturbance, tremors, dystonia, and cognitive impairment. Noch et al. reported two Chinese brothers, the older one had symptoms at the age of eight years as learning difficulties and later developed dysarthria and gait abnormalities [11]. The younger one initially had language deficits, but he developed limb ataxia and dysarthria. This phenotypic variability among patients of KRS is largely unknown, but it can be due to the degree of impairment or type of mutation in ATP13A2.

The mainstay of treatment is the symptomatic treatment of PD, with the aim of maximizing functional recovery and minimizing medication-related side effects. Levodopa has shown an important effect in the resolution of symptoms but long-term use and high doses can cause dyskinesia and motor fluctuations [12]. Further investigations on the treatment of KRS that can target metabolic derangements due to ATP13A2 mutation may provide a beneficial effect in these patients.

## Conclusions

Our case highlights the importance of diagnosing juvenile Parkinson's disease with such a rare genetic cause. KRS may present with phenotype variability, which may delay the diagnosis. However, young patients who have any kind of pyramidal and extrapyramidal symptoms must be screened for ATP13A2. Since most of these patients respond to levodopa, early diagnosis can then prevent long-term sequelae in these patients.
